# Effects of blood sample collection and preparation methods on concentrations of glucose and nonesterified fatty acids in dairy cattle

**DOI:** 10.3168/jdsc.2022-0355

**Published:** 2023-08-19

**Authors:** J.K. Drackley

**Affiliations:** Department of Animal Sciences, University of Illinois Urbana-Champaign, Urbana, IL 61801

## Abstract

Measurement of concentrations of glucose and nonesterified fatty acids (NEFA) in blood is common in nutrition and physiology studies. Proper collection and preparation conditions of the blood have been less well studied in dairy cattle. The objective of this experiment was to determine concentrations of glucose and NEFA in blood prepared with different anticoagulants (heparin vs. EDTA), use of fluoride as a glycolysis inhibitor, time until centrifugation (<30 min to 2.5 h), and plasma versus serum. Blood samples were obtained from 30 lactating cows and 15 milk-fed calves into 5 evacuated test tubes. Three of the tubes contained K_3_ EDTA and 1 tube contained heparin as anticoagulants to prepare plasma, and the fifth tube was a serum separator tube. One of the EDTA tubes was inverted and divided into 2 tubes containing NaF. One of the tubes with NaF and 1 EDTA tube were centrifuged within 30 min of collection and the others were held on ice for another 2 h before centrifugation. The heparin tube and the serum separator tube were centrifuged within 30 min. Glucose and NEFA were measured in the samples using enzymatic kits. Data were divided into 2 data sets representing normal dairy cow values or elevated values for glucose and NEFA. For glucose concentrations, results indicated that fluoride decreased concentrations, that a 2-h holding time before centrifugation did not affect results, and that serum and EDTA plasma resulted in lower glucose than heparin plasma. For low NEFA concentrations, addition of fluoride to the EDTA tubes resulted in a significant decrease of NEFA concentration. The effect of time sitting before centrifugation was significant for low NEFA samples, but contrary to our expectations, the effect was to decrease NEFA rather than increase it. Heparin as an anticoagulant did not affect NEFA concentrations in the low NEFA samples relative to EDTA. Heparin resulted in lower NEFA than serum in low NEFA samples. Serum and EDTA plasma resulted in similar NEFA concentrations. In the high NEFA samples, experimental power was limited due to the small sample size, but fluoride and time did not affect NEFA concentrations. Heparin tended to result in greater NEFA relative to EDTA. Serum produced greater NEFA values compared with EDTA but did not differ from heparin plasma. While differences among preparation methods were small and of limited biological significance, these results provide guidance for collection and processing of blood samples intended for glucose and NEFA assay.

## Highlights

• Glucose and NEFA in blood are important analytes for dairy cattle.
• Serum or anticoagulants for plasma may affect concentrations of glucose and NEFA.
• Use of EDTA plasma and serum provided lower glucose values than heparin.
• Sodium fluoride is not necessary in tubes for blood collection.
• Use of EDTA plasma is recommended for NEFA analysis.

## Graphical Abstract

**Figure:**
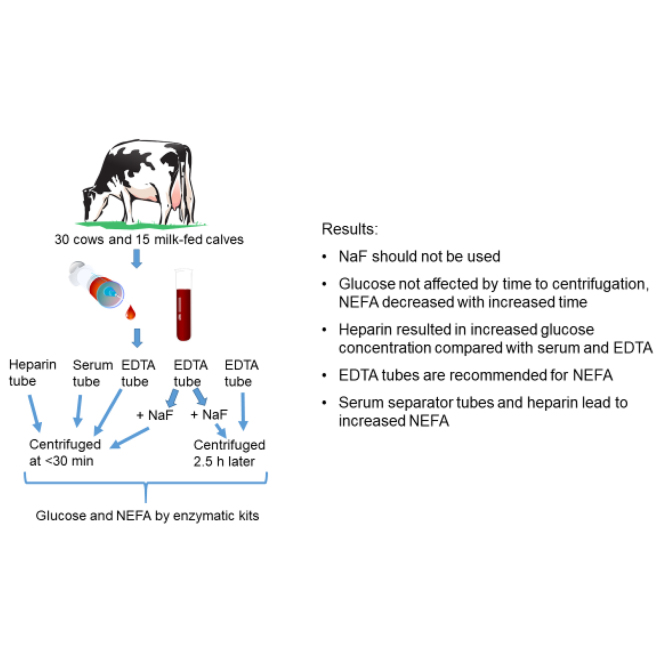
**Summary:** Measurement of glucose and nonesterified fatty acids (NEFA) is common in nutrition and physiology research. The type of blood collection and sample processing method might influence analytical values. Here, blood samples were obtained from cows and calves (n = 45) into tubes containing ethylenediaminotetraacetate (EDTA), EDTA plus sodium fluoride, heparin, or serum separator tubes. Tubes containing EDTA and EDTA plus fluoride were centrifuged immediately or held for 2.5 hours before centrifugation. While differences among preparation methods were small and of limited biological significance, these results provide guidance for collection and processing of blood samples intended for glucose and NEFA assay.

Measurement of concentrations of glucose and nonesterified fatty acids (**NEFA**) is common in nutritional and physiological studies. Commercial kits are available to measure glucose and NEFA. The choice of blood preparation method is often overlooked, and could include serum or plasma obtained by collecting whole blood in tubes with EDTA, heparin, oxalate, or citrate. A general recommendation for glucose assay has been that blood should be collected into tubes containing sodium fluoride to inhibit glycolysis by red blood cells (Sacks, 2012). Most of the recommendations made by manufacturers of assay kits came from validations with human blood. Bovine blood may not respond the same way because of the lower blood glucose concentration in ruminants and the lower use of glucose by red blood cells in ruminants (Leng and Annison, 1962). In studies with large numbers of blood samples, blood tubes may not be centrifuged for an extended time after collection, which could cause loss of glucose and an increase in lipolysis that would increase NEFA concentration. The plasma concentration of triglycerides is much lower in ruminants than in humans, providing less substrate for lipases to produce elevated NEFA. Therefore, knowledge of how different preparation methods affect measurement of glucose and NEFA concentrations in bovine blood is an important topic. Few studies have reported such measurements with bovine blood. Stokol and Nydam (2005) concluded that EDTA tubes should be used for NEFA measurements rather than heparin plasma or serum from serum separator tubes. The objective of this study was to determine concentrations of glucose and NEFA in blood prepared with different anticoagulants (heparin vs. EDTA), use of fluoride, time until centrifugation (<30 min to 2.5 h), and plasma versus serum.

The University of Illinois Institutional Animal Care and Use Committee approved all procedures with animals. Two technicians performed all procedures. Blood samples were obtained from the tail vein or artery from 30 Holstein cows and from the jugular vein of 15 milk-fed Holstein calves before the morning feeding. To obtain a wide range of NEFA and glucose concentrations, 15 cows were in early lactation and 15 cows were in mid lactation. For each sample, the blood was collected with a 20-gauge Vacutainer needle into 1 evacuated tube containing heparin (143 USP units/10-mL tube; Becton Dickinson), 3 evacuated tubes containing K_3_ EDTA (17.55 mg/10-mL tube; Becton Dickinson), and 1 evacuated serum separator tube (Becton Dickinson), in that order. Immediately after sample collection, 1 of the EDTA tubes was inverted 5 times and then divided equally between 2 test tubes containing 6.25 mg of dry NaF (weighed individually into the tubes) and again inverted to mix. All plasma tubes then were placed on ice and serum tubes were left at ambient temperature as recommended by the manufacturer. The heparin tube, 1 EDTA tube, 1 tube containing EDTA plus NaF, and the serum tube were centrifuged (1,200 × *g*) for 10 min at room temperature as soon as possible (<30 min) after collection. The remaining tubes were left on ice for 2 h and then centrifuged. The resulting plasma and serum were removed and frozen at −20°C until analysis. There was no evidence of hemolysis for any sample.

Glucose was analyzed in duplicate by the glucose oxidase method using a commercial kit (Glucose 510A; Sigma-Aldrich Chemical Co.). In the reaction, glucose oxidase oxidizes glucose to gluconic acid and hydrogen peroxide. Hydrogen peroxide reacts with o-dianisidine in the presence of peroxidase to form a colored product. Oxidized o-dianisidine reacts with sulfuric acid to form a more stable-colored product. The intensity of the pink color measured at 540 nm is proportional to the original glucose concentration. Samples were re-analyzed if the coefficient of variation for duplicate samples was >5%. The intra- and interassay coefficients of variation of a control sample were 3.9 and 4.3%.

The concentration of NEFA was determined in duplicate using a commercial kit (NEFA-C kit; Wako Fine Chemicals) using the modifications of Johnson and Peters (1993). The manufacturer recommended use of serum and stated that heparin would result in increased values. In the assay, NEFA, in the presence of ATP, CoA, and acyl-CoA synthetase, forms acyl-CoA and the byproducts AMP and pyrophosphate. Then, the product acyl-CoA is oxidized by acyl-CoA oxidase, which produces hydrogen peroxide. Peroxidase acts on the hydrogen peroxide in the presence of 3-methyl-*N*-ethyl-*N*-(β-hydroxyethyl)-aniline and 4-aminoantipyrine to form the final reaction product, a purple quinone that is detected at 550 nm. Samples were re-analyzed if the coefficient of variation for duplicates was >10%. The intra- and interassay coefficients of variation of a control sample were 5.8% and 7.2%.

The EDTA solution in the prepared tubes would dilute the analytes by 1% to 2%. Consequently, measured values were corrected for an assumed 2% dilution before statistical analysis. Addition of the dry NaF would cause a dilution of only approximately 0.13%, which was deemed negligible, and samples were not corrected. No dilution occurred with the heparin tubes since the heparin was dried on the surface of the tubes.

For all analyses, duplicate values were averaged before data analysis. Data were analyzed with a mixed effects model using Proc Mixed of SAS (version 9.4; SAS Institute Inc.). The experimental unit was the tube within cow (all 6 preparation methods). The model included the random effect of technician (n = 2) and the fixed effect of tube preparation method (n = 6). The variances for residuals for both glucose and NEFA were not homogeneous, and therefore the assumptions for ANOVA were not met. Rather than using nonparametric analyses that are more conservative, the data set was divided arbitrarily into 2 subsets for ANOVA. The subsets encompassed those with “normal” glucose and NEFA with typical concentrations for adult ruminants, and a “high” subset with high glucose (>80 mg/dL; representative of calves) and NEFA (>300 µEq/L; representative of early-lactation cows). Each subset was then analyzed using the model described above. Two extreme outliers (studentized residuals >3) were removed from the high NEFA group and the analysis was repeated. Least squares means were calculated and are presented in the tables. Least squares means were separated using 5 preplanned contrast statements as shown in the tables. Significance was declared when *P* ≤ 0.05 and trends when *P* > 0.05 but ≤0.10.

Table 1 presents the results for blood glucose determination. In contrast to our expectations, the addition of fluoride decreased glucose concentrations in the normal and high glucose samples collected into EDTA, both those centrifuged immediately and those left to sit for 2 h. A reason for the decrease is not obvious, but perhaps fluoride interfered with one or more steps in the assay. Nevertheless, our data reveal that addition of fluoride is not necessary for bovine blood and in fact may be counterproductive. Fluoride only stops glycolysis in samples maintained for more than 2 h (Bruns, 2013), so perhaps it was unlikely to influence glucose concentrations in our study.

**Table 1:** Effects of blood preparation method on glucose concentration in bovine plasma or serum^1^

Group and preparation method	Mean glucose, m*M*	SEM	Contrast, *P* <
Normal glucose, n = 30			
EDTA, spun immediately, no fluoride	3.42	0.075	Effect of fluoride, *P* < 0.001; effect of time, *P* = 0.19
EDTA, spun immediately, with fluoride	3.33	0.075	Heparin vs. EDTA spun immediately, *P* = 0.079
EDTA, spun after 2 h, no fluoride	3.49	0.075	Heparin vs. serum, *P* < 0.001
EDTA, spun after 2 h, with fluoride	3.35	0.075	Serum vs. EDTA spun immediately, *P* = 0.019
Heparin, spun immediately	3.51	0.075	
Serum, spun immediately	3.31	0.075	
High glucose, n = 15			
EDTA, spun immediately, no fluoride	5.94	0.321	Effect of fluoride, *P* = 0.002; effect of time, *P* = 0.88
EDTA, spun immediately, with fluoride	5.77	0.321	Heparin vs. EDTA spun immediately, *P* = 0.66
EDTA, spun after 2 h, no fluoride	6.00	0.321	Heparin vs. serum, *P* < 0.001
EDTA, spun after 2 h, with fluoride	5.72	0.321	Serum vs. EDTA spun immediately, *P* = 0.002
Heparin, spun immediately	5.98	0.321	
Serum, spun immediately	5.61	0.321	

1 Serum was collected in serum separator tubes.

Plasma glucose concentration was not affected by sitting for 2 h before centrifugation (Table 1). Our hypothesis was that the red blood cells would metabolize glucose during the extra time and result in lower glucose concentrations in plasma. Perhaps the lower rate of glucose use by red blood cells in ruminants (Leng and Annison, 1962) makes that less likely. Alternatively, keeping the samples on ice until centrifugation within 2 h effectively stops glycolysis with or without fluoride (Bruns, 2013). Regardless, the time of centrifugation up to 2.5 h did not affect the measured glucose concentrations.

Heparin as an anticoagulant tended (*P* = 0.08) to result in higher plasma glucose than EDTA in the normal samples, although the difference in the high glucose samples was not significant. Heparin plasma also gave greater values than serum. Serum resulted in lower glucose values than EDTA plasma in both normal and high glucose samples. Because there is no “gold standard” methodology, it is impossible to state which of these values is correct, but it is important to understand the differences among collection methods when interpreting results collected by different methods.

The author is unaware of studies that have compared bovine blood collection and preservation methods for resulting glucose concentrations. Lum and Gambino (1974) found no difference between heparin plasma and serum in humans. Mohri et al. (2008) found no difference among heparin, EDTA, and serum for glucose concentration in camels. Heparin and EDTA plasma produced similar results as serum in sheep (Mohri and Rezapoor, 2009). On the other hand, heparin resulted in significantly greater glucose concentrations than serum in ostriches (Mohri et al., 2009). Fernandez et al. (2013) found that serum and NaF-potassium oxalate plasma produced similar glucose values in samples from humans. Our results indicate that fluoride should not be used, that a 2-h holding time before centrifugation does not affect results, that serum results in lower glucose than heparin plasma, and that serum results in lower glucose than EDTA plasma.

Effects of blood collection methods on NEFA concentrations are presented in Table 2. Addition of fluoride to the EDTA tubes resulted in a significant decrease of NEFA concentration in low NEFA samples but not in high NEFA samples due to the greater variability. The effect of time sitting before centrifugation was significant for low NEFA samples, but contrary to our expectations, the effect was to decrease NEFA rather than increase it. No explanation is evident for this result.

**Table 2:** Effects of blood preparation method on concentration of nonesterified fatty acids (NEFA) in bovine plasma or serum^1^

Group and preparation method	NEFA, μEq/L	SEM	Contrast, *P* <
Low NEFA, n = 38			
EDTA, spun immediately, no fluoride	169	11.0	Effect of fluoride, *P* = 0.04; effect of time, *P* = 0.007
EDTA, spun immediately, with fluoride	162	11.0	Heparin vs. EDTA spun immediately, *P* = 0.41
EDTA, spun after 2 h, no fluoride	159	11.0	Heparin vs. serum, *P* = 0.03
EDTA, spun after 2 h, with fluoride	149	11.0	Serum vs. EDTA spun immediately, *P* = 0.17
Heparin, spun immediately	164	11.0	
Serum, spun immediately	178	11.0	
High NEFA, n = 6 or 7			
EDTA, spun immediately, no fluoride	622	138	Effect of fluoride, *P* = 0.76; effect of time, *P* = 0.47
EDTA, spun immediately, with fluoride	614	140	Heparin vs. EDTA spun immediately, *P* = 0.072
EDTA, spun after 2 h, no fluoride	551	138	Heparin vs. serum, *P* = 0.85
EDTA, spun after 2 h, with fluoride	598	140	Serum vs. EDTA spun immediately, *P* = 0.049
Heparin, spun immediately	774	138	
Serum, spun immediately	789	138	

1 Serum was collected in serum separator tubes.

Heparin as an anticoagulant did not affect NEFA concentrations in the low NEFA samples relative to EDTA but tended (*P* = 0.07) to increase NEFA in the high NEFA samples compared with EDTA. Heparin resulted in lower NEFA than serum in low NEFA samples but not in high NEFA samples. Serum produced greater NEFA values compared with EDTA in high samples but not in the low NEFA sample set. Stokol and Nydam (2005) found that serum separator tubes (as used in the current study) inflated NEFA values compared with serum obtained from a plain glass tube. They also reported that heparin increased plasma NEFA values and concluded that EDTA tubes were preferable for preparation of plasma. Similar to our study, they reported that even longer holding times (24 h) at refrigerator temperatures did not affect NEFA values (Stokol and Nydam, 2005). Studies in humans using the same analytical technique showed that NEFA concentrations were higher in serum than in EDTA plasma (McGann and Hodson, 1991; Menéndez et al., 2001). Higher values in serum have been attributed to activation of phospholipases or lipolytic enzymes during clotting or possible EDTA inhibition of the reaction (McGann and Hodson, 1991). In contrast, NEFA concentrations in sheep were nonsignificantly greater in EDTA plasma than in serum (Morris et al., 2002), as were NEFA from a study in dairy cows (Brookes et al., 1984). A reason for the discrepancies between these results and the present data is not available. Because the same analytical technique was used for these 4 studies, it seems likely that differences were related to differences in sample handling and processing.

Some sources of error variance should be recognized in this study, in addition to the analytical variation. First, the degree of dilution due to the K_3_ EDTA solution could not be measured precisely because of possible variation in the fill of the tubes during collection. Second, differences in the blood fill among multiple tubes used to assess the effect of time might have affected the degree of dilution between the samples. Collecting all blood in 1 tube, mixing it thoroughly, and then distributing it equally between the 2 time tubes would have minimized this error. However, the magnitude of error introduced by these sources likely was very small.

Because of limited sample number for the high NEFA samples, and resulting low experimental power, results for the high NEFA should be interpreted with caution. In addition, differences were small and would not in most cases lead to different interpretations of outcomes. Nevertheless, the information is important to selecting a consistent method within laboratories, and for comparing results of studies that use different preparation methods.

In conclusion, NaF is not necessary in blood collection tubes used for bovine blood centrifuged at 2 h or less after collection. Modestly extended holding times (~2.5 h) before centrifugation did not affect glucose values. Serum and EDTA plasma resulted in lower glucose concentrations than heparin plasma. For NEFA, tubes should be centrifuged as soon as possible after collection. Heparin tubes are less acceptable than EDTA tubes as an anticoagulant. Serum from serum separator tubes produced greater NEFA values compared with EDTA in both sample sets.
